# Validation of a markerless motion capture app for automated scoring of sit-to-stand, timed up and go, and short physical performance battery tests in adults with chronic disease

**DOI:** 10.1371/journal.pdig.0001172

**Published:** 2026-01-06

**Authors:** Jennifer K. Bertrand, Margaret L. McNeely, Jack Bates, Joshua Joy, Jenil Kanani, Victor E. Ezeugwu, Puneeta Tandon

**Affiliations:** 1 Department of Medicine, Faculty of Medicine and Dentistry, University of Alberta, Edmonton, Canada; 2 Department of Physical Therapy, Faculty of Rehabilitation Medicine, University of Alberta, Edmonton, Canada; 3 Department of Computing Science, Faculty of Science, University of Alberta, Edmonton, Canada; The University of Hong Kong, HONG KONG

## Abstract

Physical performance tests such as the 30-second Sit-to-Stand (30s-STS), Timed Up and Go (TUG), and Short Physical Performance Battery (SPPB) are widely used to assess physical function in older adults and are predictive of key health outcomes. However, their routine use in clinical practice is limited by time, resource, and personnel constraints. This study aimed to validate the automated scoring of physical performance assessments using a mobile, markerless motion capture (MMC) app compared to scoring by a certified exercise physiologist (CEP), and to quantify the rate and reasons for technology-related data loss. 228 adults (mean age = 61.6 ± 11.9 years) with at least one chronic medical condition were enrolled. Participants completed seven performance assessments: 30s-STS, TUG, and all components of the SPPB (Side-by-Side, Semi-Tandem and Tandem balance stands, 5-times Sit-to-Stand (5xSTS), and Gait Speed). All tests were scored simultaneously by a CEP and the MMC app using a Light Detection and Ranging (LiDAR)-enabled iPad. Agreement was assessed using intraclass correlation coefficients (ICCs) and weighted Cohen’s kappa. Agreement between the MMC app and CEP was good to excellent for all assessments. ICCs ranged from 0.812 (Tandem Stand) to 0.995 (5xSTS). The overall SPPB score showed almost perfect agreement (κ = 0.808). Perfect agreement with no variability was observed for the Side-by-Side and Semi-Tandem balance tests. The overall tech-related data loss rate was low (3.1%), with the most common issue being poor motion tracking quality (1.3%). Automated scoring of physical performance tests using a self-contained MMC app demonstrated high agreement with expert scoring and low data loss in a cohort of participants with a range of chronic medical conditions. These findings support the use of MMC-enabled mobile applications for scalable, accessible, and objective assessment of physical function in clinical settings, with future potential for remote and asynchronous use.

## Author summary

Tests of physical function—like standing up from a chair or walking a short distance—are powerful tools for understanding mobility, frailty, and future health risks in older adults. However, these tests are rarely used in routine care due to time, space, and staff constraints. In our study, we evaluated a mobile application that uses the iPad’s built-in camera to automatically measure how a person performs common movement-based tests. We tested the app with 228 older adults living with chronic illness and compared its automated scores to those from a trained health professional. We found strong agreement between the two, suggesting that accurate scoring can be achieved without a human rater. The app also performed reliably, with very few technical failures or lost data. By reducing the need for trained observers or specialized equipment, our tool could help make mobility assessments more accessible—both in clinics and at home. In future work, we aim to expand the technology for use with standard smartphone cameras and support fully self-directed testing. Ultimately, our goal is to enable broader, earlier detection of functional decline, and to support more personalized care through digital health tools that are scalable, affordable, and easy to use.

## 1. Introduction

Physical performance assessments, such as the 30-second Sit-to-Stand (STS) test [1], the Short Physical Performance Battery (SPPB) [2], and the Timed Up and Go (TUG) test [3], are essential tools for evaluating physical function in older adults, including those with chronic diseases [4]. These tests were designed to improve upon self-reported measures by providing objective and reliable insights into a patient’s physical capabilities including aerobic fitness, strength, and balance. In clinical or research settings, trained clinicians typically guide patients through these assessments and carry out direct observation and scoring. Large normative datasets support established cutoffs and performance thresholds for frailty, fall risk, and other clinical indicators [3,5–8]. These assessments are not only valuable for quantifying change with interventions (e.g., exercise) [9–11], but they are also highly predictive of outcomes such as hospitalization, disability, reduced quality of life, post-operative complications, increased mortality, and healthcare costs [2,7,12–18]. Consequently, physical performance tests are valuable not only for patients but also for clinicians and health care systems, enabling informed care and proactive decision-making [13,19].

Advances in digital technology have created new opportunities to modernize physical performance assessments, enhancing their automation, sensitivity, accessibility, and standardization. For decades, biomechanical research has leveraged digital tools for measuring physical movement, including optical tracking systems and sensors like ground force plates and inertial measurement units (IMUs) [20]. More recently, these tools have been applied to physical performance assessments, with in-depth biomechanical analyses of gait [21–23], STS [24–26], and TUG [27,28]. Most of these biomechanical studies have been conducted in controlled laboratory environments using specialized, research-grade equipment (e.g., a computerized walkway with embedded pressure sensors (GAITRite, CIR Systems, USA) [22], or a 5-sensor wearable IMU system (LegSys+, BioSensics, USA) [24]), which are costly, lab-intensive, and not well-suited for routine clinical use due to challenges in data extraction, patient burden, and workflow integration [29–32]. However, with a recognized need to use more readily available devices such as smartphone cameras to facilitate proactive health monitoring from a patient’s home [29,31], there has been an emerging intersection of biomechanical knowledge and consumer-grade technology including markerless motion capture (MMC technology).

Markerless motion capture (MMC) technology offers a low-cost and convenient solution for tracking movement during physical performance assessments. Unlike traditional marker-based motion capture systems, which require multiple near-infrared cameras and reflective markers placed on specific body landmarks [29,33], MMC leverages consumer camera technology, such as those found in smartphones and tablets, paired with advancements in computer vision and deep learning algorithms for pose estimation [34,35] These advancements have brought MMC technology closer to the accuracy levels of marker-based systems [29,33,36,37]. The move to MMC provides significant benefits, especially by reducing the time and cost associated with marker-based approaches [29,35,38], which have often been impractical for clinical settings. Beyond its cost-effectiveness and efficiency, the portability of MMC systems makes it well-suited for telehealth applications, and its rich kinematic data output creates opportunities for automated assessments and more sensitive indicators of performance.

While MMC technology has shown promise as a tool for physical performance assessments, its application in clinical research remains relatively narrow and underexplored. A 2023 review by Lam et al. found that although 65 studies applied MMC technology for clinical measurements in rehabilitation, more than half specifically focused on movement disorders such as Parkinson’s Disease and Cerebral Palsy [29]. Only a small subset of these studies assessed the validity of MMC systems for specific physical performance tests, such as shoulder range of motion [39,40]. Similarly, a systematic review on MMC technology’s use in neurodegenerative contexts revealed that most studies (24 out of 26) concentrated on gait analysis [32]. There have been efforts to automate evaluations of specific tests such as the TUG using the Xbox Kinect [41] and conventional video cameras [42–45], as well as the STS [46–50]. Despite these developments, few studies have explored the application of MMC systems for a comprehensive range of physical performance assessments or validated its automated scoring against the clinical reference standard of an expert clinician evaluation in adults with chronic illnesses.

The current study aims to validate the automated scoring of physical movement assessments using an app-based MMC system by comparing it to the clinical reference standard of scoring by a certified exercise physiologist (CEP) in a large sample of older adults with chronic medical conditions. A secondary aim is to explore the rate and reasons for MMC system-related data loss across this diverse sample.

## 2. Methods

### 2.1. Study design and population

This study is part of a larger research project investigating multidimensional contributors to the development of frailty by way of movement assessments, nutritional analysis, daily physical activity, and self-reported survey data. For this paper, only movement assessment data, self-reported survey data (e.g., demographic information), and data obtained through medical chart abstraction (e.g., Charlson Comorbidity Index) are included.

The study population consisted of community-dwelling adults recruited through local research clinics, including the Cancer Rehabilitation Clinic, the Edmonton Cirrhosis Care Clinic, and targeted social media advertisements. Inclusion criteria were: (i) a history of at least one chronic medical condition associated with increased frailty risk (condition verified by medical chart review); (ii) ≥18 years of age; (iii) ability to perform functional movement assessments, wear a smartwatch for one week, and log dietary intake for two days; and (iv) capacity to provide informed consent. All research activities were approved by the University of Alberta’s Health Research Ethics Board (Pro00117501), and all participants provided written informed consent prior to participation.

### 2.2. Procedure

The study was conducted at the University of Alberta in Edmonton, Alberta, Canada, between December 2023 and August 2024. Movement assessments were conducted in a clinical laboratory setting. Initially, the first 32 participants completed the test battery over two separate laboratory visits spaced 6–14 days apart, with approximately half of the assessments performed at each visit. Following protocol refinement to simplify logistics and minimize participant burden, all subsequent participants completed the full test battery in a single laboratory visit, with a scheduled mid-session rest period to reduce fatigue and ensure participant comfort.

Prior to the clinic visit, participants completed an online consent form, a demographic survey, and the Physical Activity Readiness Questionnaire (PAR-Q). Upon arrival, their PAR-Q responses were reviewed by a certified exercise physiologist (CEP) experienced in working with individuals with chronic medical conditions. The review included discussions about limitations affecting their ability to complete the movement assessments, review of blood pressure and heart rate, and participants were invited to opt out of any tests for which they felt they would experience discomfort. Prior to beginning the movement assessments, clearance for physical activity was determined by the CEP, with as needed, but infrequent physician consultation.

#### 2.2.1. Movement assessments.

During their visit, participants performed a series of movement assessments under the instruction of the CEP. The assessments included the 30-second Sit-to-Stand (30s-STS) test [1], the Timed Up and Go (TUG) test [3], and the 5 tests of the Short Physical Performance Battery (SPPB) [2]: the Side-by-Side Stand, the Semi-Tandem Stand, the Tandem Stand, the 5-times Sit-to-Stand (5xSTS) test (corresponding to the ‘Repeated Chair Stand Test’ described in the original SPPB validation [2]), and the Gait Speed test. These assessments were selected for their widespread use in clinical practice and research, with large normative datasets that establish population-based reference values for each test [2,51,52], systematic reviews documenting their psychometric properties [53–59], and consistent inclusion in geriatric assessment guidelines [60–62].

The tested movement assessments are further detailed in Table 1. The SPPB total score was calculated following the SPPB scoring protocol [2], with the five constituent test scores summed to yield a total score between 0 (lowest function) and 12 (highest function). To ensure participant safety, for the Side-by-Side, Semi-Tandem, and Tandem Stands, participants were offered the option of having a chair placed within arm’s reach for support if balance was lost.

**Table 1:** Description of movement assessments and MMC system setup.

Assessment Name	Assessment Description	Clinician Measurement Protocol	Measurement Type	MMC System Setup (iPad Placement)
**30-second Sit-to-Stand (30s-STS)** [1]	Repeated sit-to-stand movements from a chair over 30 seconds	Count number of full upright stands completed within 30 seconds	Continuous (0–X reps complete)	Camera placed at front-right angle to participant and chair, ~ 2.5 m distance
**Timed Up and Go (TUG)** [3]	Stand from chair, walk 3 meters, turn, return, and sit down again	Time to complete full sequence	Continuous (s)	Camera placed in front of participant, ~ 5 m from starting position/chair
**SPPB: Side-by- Side Stand** [2]	Standing with feet together for up to 10 seconds	Time held (up to 10 seconds)	Continuous (0–10s)	Camera placed directly in front of participant, ~ 2.5 m distance
**SPPB: Semi-Tandem Stand** [2]	Standing with one heel of one foot next to the instep of the other for up to 10 seconds	Time held (up to 10 seconds)	Continuous (0–10s)	Camera placed to side of participant, ~ 2.5 m away, with back foot closest to camera
**SPPB: Tandem Stand** [2]	Standing with one foot directly in front of the other for up to 10 seconds	Time held (up to 10 seconds)	Continuous (0–10s)	Camera placed to side of participant, ~ 2.5 m away
**SPPB: 5-times Sit-to-Stand (5xSTS)** [2]	Rising from a seated position to standing and sitting again, 5 times, as quickly as possible	Time to complete 5 stands	Continuous (s)	Camera placed at front-right angle to participant and chair, ~ 2.5 m distance
**SPPB: Gait Speed** [2]	Walking 3 meters at a usual pace	Time to walk 3 meters, divided by 3 meters	Continuous (s)	Camera placed in front of participant, ~ 5 m from start line
**SPPB: Total Score** [2]	Composite score summing results from balance tests, Gait Speed, and 5xSTS	Calculated with established scoring protocol (range: 0–12), sum of 5 component test scores	Ordinal (0–12)	Derived from video recordings of individual tasks; MMC algorithms apply scoring logic per test

Because the objective of this study was to compare scoring agreement between a MMC system and clinician measurement, and not to assess change or performance differences across conditions or sessions, randomization of assessment order was not required. A fixed order of assessments was implemented to align with clinical workflows and optimize participant comfort, with a 15-minute break (used for instructions on other study elements) scheduled halfway through the tests.

#### 2.2.2. The Ameya MMC App.

**Fig 1:**
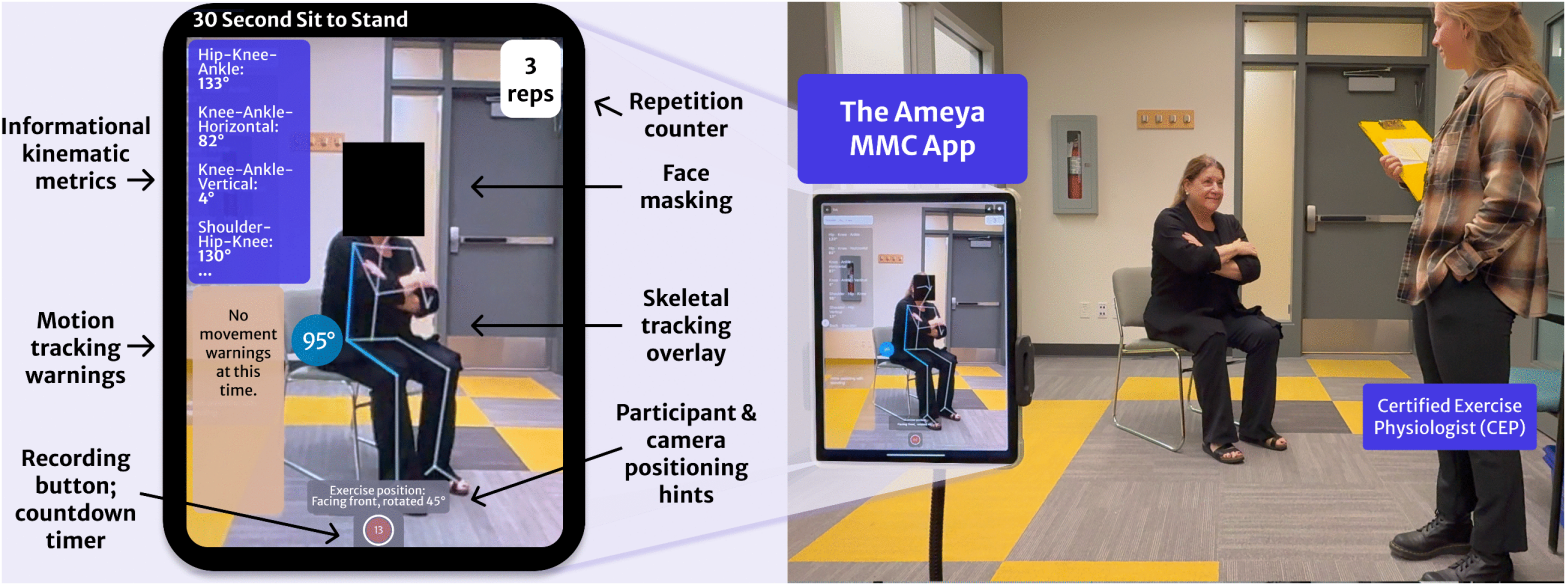
Ameya MMC App and in-clinic setup with the CEP.

A custom-built iOS application, the Ameya MMC app (Fig 1), was developed for this study to support multidimensional frailty assessment, including automated scoring of physical performance tests (Tables 1 and 2) using MMC technology. The app captures 3D joint position data from 34 anatomical landmarks at approximately 30 Hz using the Light Detection and Ranging (LiDAR) depth-sensing camera built into certain iPad Pro and iPhone Pro models.

**Table 2:** Summary of test-specific algorithm logic and output metrics.

Assessment Name	Input Signal	Core Detection Logic	Output Metric(s) Displayed in App
**30-second Sit-to-Stand (30s-STS)** [1]	Vertical body motion	Detect repeated sit-to-stand transitions over a fixed 30-second period; apply movement thresholds and filters to avoid false counts.	Number of full stands completed; data quality flag.
**Timed Up and Go (TUG)** [3]	Body-height change during sit-to-stand and return-to-sit	Detect stand-up and sit-down events from body-height transitions; compute time between leaving and returning to the seated position.	Total time (seconds); data-quality flag.
**SPPB Balances (Side-by-Side, Semi-Tandem, Tandem)** [2]	Hand and foot/ankle motion relative to start position	Detect loss of balance using hand or foot movement.	Duration held (up to 10 seconds); pass/fail balance score; data-quality flag.
**SPPB: 5-times Sit-to-Stand (5xSTS)** [2]	Vertical body motion	Identify five complete sit-to-stand cycles; measure total time between the first and fifth stands.	Time to complete five stands (seconds); data-quality flag.
**SPPB: Gait Speed** [2]	Forward body (core) displacement	Estimate forward walking speed from body motion over 3 meters; verify consistency across partial-distance segments	3-meter gait duration (seconds) and derived score; data-quality flag.

The app’s MMC component is built using a skeletal tracking module which supports both RGB and RGB-D camera input. In this study, real-time pose estimation was performed using the LiDAR-based depth camera on the iPad Pro (Fifth/Sixth generation, Apple Inc., US). The Ameya MMC app incorporated this tracking framework to enable automated, test-specific scoring procedures for the investigated physical performance assessments. Participants were assigned individual profiles within the app, which allowed trained research assistants (RAs) to initiate the appropriate assessments using the clinician-facing interface.

A consistent setup protocol was used during data collection (iPad placed in a tripod approximately 2.5 m away from the participant, with the camera lens approximately 0.5 m off the ground; see Table 1 and Fig 1), however, assessments were conducted in multiple clinical rooms with variation in flooring, lighting, and background. The tracking system operated robustly under these conditions without additional configuration or environmental controls.

Test-specific scoring algorithms were executed in real time during each assessment (see Table 2). Each algorithm was tailored to the corresponding movement test, where movement signals like vertical displacement or forward motion were used to identify key events like stand transitions, walking phases, or balance loss. These scoring algorithms included repetition detection and counting for the 30s-STS test and the 5xSTS test, time estimation for the TUG test and the Gait Speed test, and duration thresholds for balance tests. Output metrics were calculated automatically and displayed immediately on the app interface after each test. MMC app recordings were stored locally on the iPad and automatically synchronized with a secure cloud server, and included in-app face masking (see Fig 1) for enhanced participant privacy.

The application also included error detection and interface elements to support use by non-technical staff. These included countdown timers, real-time movement counters, and visual indicators when pose estimation was compromised (e.g., due to occluded joints or camera misalignment).

During pilot testing, scoring algorithms were tested and refined to accommodate varied movement speeds, body sizes, and compensatory postures observed in the target population. Subsequent minor adjustments to the scoring algorithms were performed iteratively during data collection in response to observed edge cases, optimizing smoothing parameters for variable joint trajectories such as irregular repetitions, and increasing tolerance to partial occlusions when limbs were temporarily obscured. These refinements did not alter the underlying scoring logic but improved the robustness of detection across participants. All recordings were reprocessed at the conclusion of the study’s data collection period using the finalized algorithm set (one per movement test) to ensure scoring consistency across the full dataset.

Altogether, the Ameya MMC app’s architecture includes real-time pose tracking, task-specific scoring logic, automated data handling, and built-in quality control mechanisms. These components were developed to support standardized and scalable assessment of physical performance in structured clinical research settings.

#### 2.2.3. Additional measurements.

Outside of the in-person session, participants completed additional questionnaires via REDCap, including a demographic survey, the revised Edmonton Symptom Assessment Scale [63], the Edmonton Frail Scale [64], the EQ-5L-5D [65], the Duke Activity Status Index (DASI) [66], and a fall history survey. For participants who gave explicit consent (n = 226), medical chart abstraction was performed to obtain clinical data (e.g., history of chronic disease or cancer) and calculate the Charlson Comorbidity Index (CCI) [67]. The CCI is a measure of comorbidity burden, assigning weighted scores to chronic conditions, with higher scores indicating increased comorbidity and mortality risk.

### 2.3. Outcomes

The primary outcome was the level of agreement between the Ameya MMC app and a clinician (CEP), evaluated separately for each of the seven movement assessments, as well as for the overall SPPB score. Agreement was assessed with Intraclass Correlation Coefficients (ICCs) for the following metrics: (i) TUG duration (seconds); (ii) number of repetitions in the 30s-STS test; (iii-v) balance duration (up to 10 seconds) for the Side-by-Side, Semi-Tandem, and Tandem Stands; (vi) time (seconds) to complete five sit-to-stand repetitions; and (vii) walking speed during the Gait Speed test. Agreement for the SPPB total score (range: 0–12) was also assessed.

The rate and reasons for tech-related data loss per movement assessment and overall were tracked. Tech-related data loss was defined as the percentage of MMC app recordings that were not analyzable, omitting participant or protocol-related exclusions.

Four types of tech-related issues were identified: (i) app crashes (any instance where the MMC app failed after recording had begun), (ii) incomplete data (occurred where earlier versions of the real-time algorithm prematurely terminated a test before the completion criteria was met), (iii) poor motion tracking quality, determined through visual inspection (e.g., skeletal overlay misaligned with participant joints), field notes (e.g., tracking of background objects was observed by the iPad operator), or detection of implausible joint movements (e.g., multi-joint velocity spikes = faster than 5 m/s, a conservative cutoff well above expected speeds for these clinical tasks [24,27,68–72]), and (iv) stand count discrepancies with the 5xSTS test (the CEP counted five repetitions but the MMC system did not, leading to the manual stopping of recordings that prevented the MMC app from calculating a duration).

### 2.4. Statistical analysis

Descriptive statistics were calculated using appropriate summary measures for continuous and ordinal outcomes. Agreement between the CEP and MMC app on continuous test outcomes was assessed using ICCs with 95% confidence intervals, calculated using single-rating, absolute-agreement, two-way mixed-effects models (ICC(2,1)) following guidelines from Koo and Li [73]. ICC values were interpreted using the following thresholds: values below 0.5 were considered to reflect poor agreement, values between 0.5 and 0.75 indicated moderate agreement, values between 0.75 and 0.9 indicated good agreement, and values greater than 0.9 indicated excellent agreement [73]. ICCs were undefined when no between-subject variability was present.

Agreement on the discrete, ordinal SPPB total score (range: 0–12) was evaluated using a weighted Cohen’s kappa with quadratic weights. This method accounts for agreement by chance and penalizes larger differences more heavily. Kappa values were interpreted based on the classification system by Landis and Koch [74]: values below 0.00 were considered poor agreement; values between 0.00 and 0.20 slight; 0.21 to 0.40 fair; 0.41 to 0.60 moderate; 0.61 to 0.80 substantial; and 0.81 to 1.00 almost perfect agreement. All analyses were conducted using Jamovi (version 2.3) and the ‘seolmatrix’ module [75] based on the ‘irr’ package [76] in R.

## 3. Results

### 3.1. Characteristics of the participants

A total of 228 adults with at least one chronic medical condition or a history of cancer were enrolled. Demographic characteristics are summarized in Table 3. Of the total sample, 62% were female, 79% identified as white, and mean age was 61.6 ± 11.9 years. The cohort demonstrated a high burden of chronic disease and comorbidity, with a mean Charlson Comorbidity Index (CCI) score of 4.52 ± 2.37. The most commonly reported chronic conditions were a history of cancer (37%) and cirrhosis (25%). Additional diagnoses (grouped into the Other category in Table 3 Disease History) spanned a broad range, including neurological conditions (e.g., Parkinson’s disease, multiple sclerosis), post-transplant status, inflammatory arthritis, and diabetes.

**Table 3:** Participant characteristics.

Participant Characteristics	*n* (%) or *M *± *SD*
Age (years)	61.6 ± 11.9
Sex	
Female	142 (62.3)
Male	86 (37.7)
Gender (n = 227)	
Woman	139 (61.2)
Man	87 (38.3)
Other	1 (<1)
Race (n = 224)	
White	177 (79.0)
East Asian	12 (5.4)
South Asian	10 (4.5)
Aboriginal	6 (2.7)
Latino	4 (1.8)
Middle Eastern	2 (0.9)
Black	1 (<1)
Other	4 (1.8)
Mixed race	8 (3.6)
BMI (kg/m²) (n = 226)	29.2 ± 6.6
Disease History* (n = 226)	
Cancer	84 (37.2)
Cirrhosis	57 (25.2)
Other	103 (45.6)
Charlson Comorbidity Index (n = 226)	4.52 ± 2.37
Gait Aid Used (n = 218)	16 (7.3)
Falls 1 year prior (n = 218)	
None	135 (61.9)
One	45 (20.6)
Two	20 (9.2)
More than two	18 (8.3)
Duke Activity Status Index score (n = 212)	42.7 ± 12.3
EQ-5D-5L VAS rating (n = 218)	68.9 ± 18.4
Edmonton Symptom Assessment Scale (n = 210)	
Pain	2.49 ± 2.19
Tiredness (lack of energy)	3.72 ± 2.68
Drowsiness (feeling sleepy)	2.70 ± 2.53
Nausea	0.50 ± 1.22
Appetite	3.28 ± 2.76
Shortness of breath	1.50 ± 2.26
Depression	1.64 ± 2.33
Anxiety	1.90 ± 2.29
Wellbeing (overall feeling)	3.69 ± 2.70
Edmonton Frail Scale score (n = 218)	
Fit (score 0–3)	102 (46.8)
Vulnerable (score 4–5)	53 (24.3)
Mild Frailty (score 6–7)	40 (18.3)
Moderate Frailty (score 8–9)	16 (7.3)
Severe Frailty (score 10+)	7 (3.2)

N = 228 unless otherwise indicated. Sample sizes vary due to missing data. *Categories are not mutually exclusive; participants may have multiple conditions.

Participants reported moderate functional capacity in daily life, with a mean DASI score of 42.7 ± 12.3 (n = 212). Overall health status, as rated on the EQ-5D-5L visual analog scale, averaged 68.9% ± 18.4% (n = 218). A gait aid was regularly used by 7% of the participants, and 38% reported at least one fall in the previous year. Symptom burden, as measured by the Edmonton Symptom Assessment Scale (n = 210), was generally low, with the highest mean scores observed for tiredness (3.72 ± 2.68), overall wellbeing (3.69 ± 2.70), and appetite concerns (3.28 ± 2.76). Based on the Edmonton Frail Scale (n = 218), 46.8% were categorized as not frail, while 24.3% were classified as vulnerable, and 25.6% with mild to moderate frailty. Seven participants (3.2%) were classified as severely frail.

### 3.2. Agreement between CEP and the MMC app

Agreement between the outcomes from the CEP and the MMC app (Table 4) was excellent for the TUG [ICC = 0.96, 95% CI: 0.93–0.98], the 30s-STS [ICC = 0.93, 95% CI: 0.90–0.95], and the 5xSTS test [ICC = 0.995, 95% CI: 0.98–0.99]. Agreement on Gait Speed was also high [ICC = 0.87, 95% CI: 0.80–0.91].

**Table 4:** Agreement between MMC and clinician (CEP).

Movement Assessment	Outcome	*n*	ICC (95% CI)	Cohen’s kappa
**Timed Up and Go (TUG)**	Duration (s)	226	**0.962** (0.927, 0.977)	
**30-second Sit-to-Stand (30s-STS)**	Count (reps)	222	**0.928** (0.895, 0.949)	
**SPPB: Complete Battery**	Score (0–12)	178		**0.808**
** Side-by-Side Stand**	Duration (0–10s)	220	perfect agreement (ICC undefinable*)	
** Semi-Tandem Stand**	Duration (0–10s)	220	perfect agreement (ICC undefinable*)	
** Tandem Stand**	Duration (0–10s)	200	**0.812** (0.758, 0.855)	
Tandem Stand (hand support excl.**)	Duration (0–10s)	194	**0.979** (0.972, 0.984)	
**SPPB: 5-times Sit-to-Stand (5xSTS)**	Duration (s)	213	**0.995** (0.983, 0.998)	
**SPPB: Gait Speed**	Speed (m/s)	226	**0.868** (0.796, 0.910)	

*There was perfect agreement and no variability within participants for the Side-by-Side and Tandem Stands, so an ICC value could not be computed for these two movement assessments.

**Additional exclusions on Tandem Stand are the removal of 6 cases where participants braced themselves with the back of a chair that was placed beside them for safety.

For both the Side-by-Side and Semi-Tandem Stands, the MMC app recorded perfect agreement with the CEP. However, ICCs were undefined due to a ceiling effect; all participants completed the full 10-second duration, resulting in no between-subject variability.

Of all tests, the Tandem Stand showed the lowest agreement between the CEP and the MMC app [ICC = 0.81, 95% CI: 0.76–0.86], though the value remained in the good range. Further inspection revealed six instances in which participants lost balance and used a nearby chair for support. While the CEP recorded this as the end of the test, the MMC app was unable to detect the chair support as it occurred behind the patient and out of the field of view of the camera. When these six cases were excluded, agreement improved to excellent [ICC = 0.98, 95% CI: 0.97–0.98].

For the total SPPB score, calculated as the sum of the individual SPPB components, agreement between the CEP and the MMC app was high, with a weighted Cohen’s kappa of 0.808, indicating almost perfect agreement.

### 3.3. Tech-related data loss

Tech-related data loss was tracked across the seven movement assessments and is summarized in Table 5. After removing non-technical exclusions (e.g., participant opt-outs, protocol deviations), 1576 total MMC app recordings remained. Of these, 1527 were analyzable, resulting in a tech-related data loss rate of 3.1% (49 recordings).

**Table 5:** Participant exclusions and MMC app data loss.

		Side-by-Side Stand	Semi-Tandem Stand	Tandem Stand	Gait Speed	5xSTS	30s-STS	TUG	All Tests Combined
**N Total Participants**		**228**	**228**	**228**	**228**	**228**	**228**	**228**	**1596**
*Non-Tech Exclusions*									
Participant Opt-Out				2		7	2		11
Protocol Deviation			1	6			2		9
**N After Non-Tech Exclusions**		**228**	**227**	**220**	**228**	**221**	**224**	**228**	**1576**
*Tech Exclusions*									
App Crash		2	2	3			2	1	10
Incomplete Data (Early Algorithm)		4		5		3		1	13
Poor Motion Tracking Quality		2	5	12	2				21
Stand Count Discrepancy						5			5
**N After Tech Exclusions**		**220**	**220**	**200**	**226**	**213**	**222**	**226**	**1527**
**% Tech-Related Data Loss**		**3.5**	**3.1**	**9.1**	**0.9**	**3.6**	**0.9**	**0.9**	**3.1**

Of the small number of excluded recordings (49 of 1576 total recordings), the most common reason for exclusion was poor motion tracking quality (21 recordings, or 1.3% of all MMC app recordings). Other exclusion reasons included incomplete data due to errors in early algorithm versions (13 recordings) and app crashes (10 recordings), though each type of exclusion amounted to less than 1% of data loss.

Three assessments—Gait Speed, 30s-STS, and TUG—had minimal tech-related data loss (<1%). The 5xSTS test had a specific issue related to stand count discrepancies. In five cases, MMC detected only four repetitions, while the CEP identified five. Because the MMC app’s stopping condition was not met, the duration could not be calculated and agreement could not be assessed.

The Tandem Stand had the highest tech-related data loss rate, with 20 unusable MMC app recordings (9% of recordings for that test). Most of these were due to poor motion tracking (12 recordings). The algorithm struggled with depth cues and differentiating foot placement when participants wore bulky clothing (e.g., long shorts, sweatpants) or dark-colored attire. These issues and potential solutions are elaborated on in the Discussion.

## 4. Discussion

This study validated the use of the Ameya MMC app for automated scoring of physical performance tests in a sample of 228 adults with chronic disease. Agreement between gold-standard clinician-determined scores and app-based MMC system scores was assessed for the 30-second STS test, the TUG test, and all 5 tests of the SPPB. Good to excellent agreement was observed across all assessments, with the TUG, 30s-STS and 5xSTS tests yielding ICC values ranging from 0.928 to 0.995. Clinician and MMC app agreement on the overall SPPB score (composite score of 5 summed scores) was found to be almost-perfect (weighted Cohen’s kappa of 0.808). Across all seven physical performance tests, only two tests, the Tandem Stand and Gait Speed, were below the threshold for excellent agreement, with ICCs of 0.812 and 0.868, respectively. Overall, these results demonstrate that with the use of carefully developed algorithms, app-based scoring of in-clinic physical performance assessments using the Ameya MMC app provides good to excellent agreement with gold-standard clinician-determined scoring.

Despite strong evidence linking physical performance tests to clinical outcomes such as hospitalization, disability, and mortality [12–17,77,78], gold-standard, clinician-determined assessments remain underutilized in routine care, including in rehabilitation and chronic disease management [79–81]. Across care settings, clinicians cite time constraints, lack of trained or certified personnel, and other practical limitations as barriers to administering these standardized and objective tests [82–85]. The current study begins to address these challenges by demonstrating that physical performance assessments can be accurately scored using a self-contained app running on an iPad, eliminating the need for external sensors, computing infrastructure, or post-testing analysis. This easy-to-use setup lowers the barrier to in-clinic use and suggests the potential of future remote, asynchronous use, expanding access for patients and scalability for health systems.

Others have explored the supplementary use of consumer-grade technology and MMC technology with physical performance assessments, using the Microsoft Kinect or other simple RGB cameras for motion tracking, however, these approaches typically require additional computing infrastructure, technical expertise, and are often limited to a single assessment type [29]. In contrast, the current study is, to our knowledge, the first to demonstrate expert-level scoring across a suite of standardized physical performance tests using a standalone, real-time mobile app. This includes seven validated tests administered during a single clinical session across a large and clinically diverse population of individuals with at least one chronic condition predisposing to frailty. Although previous studies have tested large samples and/or multiple performance tasks [86,87], none have combined portability, real-time scoring, clinical usability, and a broad range of validated assessments, within a single, plug-and-play app compatible with any LiDAR-enabled device (e.g., iPhone Pro or iPad Pro models). This tool may help address practical barriers to physical performance assessment in clinical settings by offering an accessible and standardized measurement approach.

Building on this unique approach, these findings can be situated alongside recent efforts to automate physical performance assessments using non-laboratory, MMC technology-based tools. In a series of studies [88–90], an electronic SPPB kiosk demonstrated excellent agreement with manually scored assessments (ICC = 0.92 for the total score [90]). While the kiosk also used LiDAR sensor technology, it incorporated additional hardware, including a load cell array (weight-sensitive mat) for the three balance tests and the 5xSTS [90]. Despite this added hardware, its scoring precision was comparable to or lower than that of the current study. For instance, the balance ICC (0.89, 95% CI: 0.81–0.93) and 5xSTS ICC (0.84, 95% CI: 0.75–0.90) [90] were similar to or lower than those observed here (0.81 for Tandem Stand before exclusions, and 0.99 for 5xSTS). Their data loss rate (3.3%, defined as the proportion of participants excluded due to incomplete data capture [90]) was also similar to that observed in the present study.

In another large-scale study, Liu et al. [86] tested 665 older adults using a Microsoft Kinect, custom software, a Mini PC and a custom TV-based interface. Their system incorporated seven functional assessments, including STS, TUG, and Gait Speed, and produced correlation coefficients between automated and clinician scores. Most correlations were strong and consistent with the current study findings, except for Gait Speed, where their correlation (r = 0.493) showed much weaker agreement than our measure of agreement (ICC = 0.868). Data loss was not reported. In a much smaller sample of 30 older adults, Kaewakaen et al. [91] achieved excellent agreement with an expert rater using a Kinect for the 5STS, further supporting the feasibility of automated scoring in this domain. Together, these comparisons illustrate the promise of prior MMC systems but also underscore the benefits of a streamlined, app-based approach.

These findings support the use of the Ameya MMC app as a practical tool to facilitate accurate, objective physical performance testing in clinical settings. Rather than replacing clinician expertise, the app is designed to complement it, reducing scoring burden while maintaining accuracy. Advantages include automated scoring, improved efficiency, and the potential for non-specialized staff to conduct assessments, which may enable more frequent monitoring of physical function [31,32].

The tool may be particularly relevant in resource-limited settings [29,92], such as rural clinics or long-term care facilities, where access to trained assessors is often constrained [31]. In these contexts, it could support earlier identification of deficits that may be modifiable with intervention. In research contexts, the automated evaluation through the app could also streamline data collection and make it more feasible to include physical performance testing as an objective outcome measure across larger or more distributed studies. By facilitating accurate, scalable, and accessible assessment [29,31,32], this approach supports more equitable, patient-centered models of care [93,94] and serves as a promising adjunct to traditional in-person assessments.

### 4.1. Limitations

While the automated MMC scoring approach demonstrated strong overall agreement, several limitations and design considerations emerged. One key challenge was validating this MMC app approach against clinician judgment, which includes a degree of subjectivity. For example, CEPs may have counted a borderline STS repetition that occurred just after the timer ended or have allowed for slight deviations in form (such as not standing fully upright), whereas the MMC app’s rigid nature with fixed thresholds did not accommodate these nuances. This occasionally led to discrepancies, such as the MMC app scoring only four repetitions when the CEP counted five, highlighting a trade-off between algorithmic precision and assessor flexibility. CEPs also relied on the MMC app’s auditory countdown to synchronize their stopwatch, likely introducing small timing inconsistencies due to reaction time, which may have added noise to the reported agreement measures.

The Tandem Stand posed the greatest challenge in terms of both scoring accuracy and data quality. For safety, the test requires a nearby chair for support, and the test is stopped if the participant uses the chair. While CEPs could directly observe whether support of the chair was used, the MMC app could not detect this, as the chair was often occluded from the camera’s view. Modifications to the algorithm that account for this scenario are underway. Exclusion of these six cases substantially improved agreement. Beyond this challenge, the static nature and sagittal orientation of the Tandem Stand reduced the MMC app’s ability to detect and track positions of the key body joints, especially in the lower body, leading to poor tracking in some participants. These issues were most common when participants wore dark or loose-fitting clothing or footwear that obscured joint landmarks. Future improvements may improve participant positioning and algorithmic handling of common occlusion scenarios.

Gait Speed measurement also presented unique challenges, as it required participants to move over a longer distance than the other tests. Unlike CEPs, who timed a marked 3-meter section, the MMC app does not track floor markers and instead relies on depth measurements that may not be captured over an identical time window to the CEP. This approach introduced some variability, particularly given the LiDAR sensor’s reduced depth accuracy beyond 5 meters—an issue that occasionally affected tracking quality when participants were furthest from the iPad (at the start of their Gait Speed test).

Because both the MMC app and the CEP were scoring the same test instances, participant instructions were delivered solely by the CEP, and the app included only a countdown timer. Observing the instructional flow and challenges across a large sample has helped inform the design of automated instructions to be integrated in future app versions, similar to the approach taken by Liu et al. [86]

Beyond these procedural considerations, sample composition also presents limitations in this study. Approximately 80% of participants identify as White, and the cohort is largely composed of older adults. This limits generalizability to younger populations with chronic disease and to individuals from diverse racial and ethnic backgrounds. Prior work has shown that computer vision systems may perform less accurately across different skin tones and body types [95,96], and broader digital health research warns that inadequate diversity in training and validation can perpetuate health inequities [97–99].

Finally, these findings are currently limited to supervised, in-clinic use and are not yet generalizable to remote or unsupervised settings. The current approach also depends on LiDAR-enabled devices, which are typically newer, higher-end consumer models—potentially limiting accessibility for some users. Taken together, these limitations illustrate important design considerations that will guide further app refinement to improve usability, performance, reliability, and generalizability.

### 4.2. Future directions

Building on the insights from this study, next steps will focus on expanding the app’s utility beyond supervised, in-clinic use. This includes integrating automated instructions to guide participants through assessments without a clinician present, refining test algorithms to better handle edge cases, especially those stemming from safety considerations (e.g., detecting compensatory arm movements during balance tests), and adapting assessments so they could be used with 2D/non-LiDAR smartphone cameras (e.g., with Android phones or tablets, and non-Pro iOS models). Real-time tracking quality checks will be explored to flag cases where tracking is compromised—such as due to dark or loose clothing—allowing participants to correct the issue before data is recorded (and thus reducing the rate of data loss to poor motion tracking).

The MMC app is being adapted for remote and asynchronous use, allowing users to perform assessments on their own at home. Doing so requires thoughtful updates, not just to the technology (e.g., adjustments to enable non-LiDAR, 2D smartphone camera use), but also to safety protocols and user interface design, especially to support older adults or individuals with limited mobility. We envision a future where a simple 30s-STS task, captured with a tablet in a clinic or home setting, becomes a routine part of health monitoring, just like blood pressure, weight, or height [12,100,101]. Just as automated blood pressure cuffs have enabled non-clinicians to reliably capture vital signs, advances in MMC have the potential to bring the same level of objectivity and convenience to the assessment of physical function.

In parallel, the next steps will explore how the rich kinematic data captured during app-based MMC assessments could yield deeper clinical insight—without placing any extra burden on patients or clinicians. These continuous movement signals, already collected by the MMC app, may offer a more nuanced and objective view of how these tasks are performed, beyond the final score. For example, two individuals might complete 12 repetitions in a 30-second STS test, but one performs with fluid, controlled motion while the other shows erratic pacing or compensatory strategies that could signal instability, muscular deficits, disease, or fall risk [24,26,70,102–104]. Reducing the performance metrics into a single repetition count or duration obscures meaningful differences in movement quality [32,105,106]. By utilizing the full potential of kinematic data from MMC technology, we can enhance clinical interpretation, identifying subtle impairments that traditional scoring methods may miss. This represents an important step to maximizing the value of digital assessments—moving from simple tools for quantifying gross test performance to a platform that provides deeper insights into functional ability. These next steps are intended to advance the integration of physical function monitoring into both research and routine care in a more consistent and accessible manner.

## 5. Conclusion

This study validated a self-contained, app-based MMC system for scoring physical performance assessments in a large and clinically diverse sample of adults with chronic medical conditions. Good to excellent agreement was found between the app’s automated scores and those of a trained clinician (CEP) across multiple functional tests, supporting its accuracy and feasibility for in-clinic use. By enabling automated scoring within a portable app, this approach may help to reduce barriers to standardized physical function assessment. With continued development in this field, there is potential to support more scalable, accessible, and patient-centered approaches to frailty and fall-risk screening, prehabilitation and tracking rehabilitation progress, as well as proactive monitoring of physical, functional health across the lifespan.

## Supporting information

S1 Table Average scores recorded by CEP and MMC app for each movement assessment. (PDF)

S1 Dataset Per subject data for each physical performance test. (ZIP)
