# Supplementary material for: Validation of a markerless motion capture app for automated scoring of sit-to-stand, timed up and go, and short physical performance battery tests in adults with chronic disease
Source: PLOS Digit Health. 2026 Jan 6;5(1):e0001172. doi: 10.1371/journal.pdig.0001172 (PMC12773808; doi:10.1371/journal.pdig.0001172)
Supplement: S1 Table — (PDF) [file pdig.0001172.s001.pdf]

S1 Table. Average scores recorded by CEP and MMC app for each movement assessment

Movement Assessment	Outcome	*n*	CEP (Mean ± SD)	MMC App (Mean ± SD)
**Timed Up and Go**	Duration (s)	226	9.94 ± 2.55	10.2 ± 2.31
**30 Second Sit to Stand**	Count (reps)	222	11.5 ± 4.06	11.1 ± 3.93
**SPPB - Complete Battery**	Score (0-12)	178	10.9 ± 1.48	11.0 ± 1.36
**Side-by-Side Stance**	Duration (0-10s)	220	10.0 ± 0.0	10.0 ± 0.0
**Semi-Tandem Stance**	Duration (0-10s)	220	10.0 ± 0.0	10.0 ± 0.0
**Tandem Stance**	Duration (0-10s)	200	9.69 ± 1.41	9.81 ± 1.05
**Tandem Stance** *(hand support excl.*)*	Duration (0-10s)	194	9.84 ± 1.06	9.86 ± 0.925
**SPPB - 5 Chair Stand**	Duration (s)	213	11.3 ± 4.14	11.5 ± 4.07
**SPPB - Gait Speed****	Duration (s) to complete 3m	226	3.34 ± 0.885	3.17 ± 0.801

**Additional exclusions on Tandem Stance are the removal of 6 cases where participants braced themselves with the back of a chair that was placed beside them for safety.*
